# Effect of phlebotomy versus oral contraceptives containing cyproterone acetate on the clinical and biochemical parameters in women with polycystic ovary syndrome: a randomized controlled trial

**DOI:** 10.1186/s13048-019-0554-9

**Published:** 2019-08-30

**Authors:** Samira Behboudi-Gandevani, Hayedeh Abtahi, Navid Saadat, Maryam Tohidi, Fahimeh Ramezani Tehrani

**Affiliations:** 1 Reproductive Endocrinology Research Center, Research Institute for Endocrine Sciences, Shahid Beheshti University of Medical Sciences, No 24, Parvane Street, Yaman Street, Velenjak, P.O.Box: 19395-4763, Tehran, Iran; 2 Endocrine Research Center, Research Institute for Endocrine Sciences, Shahid Beheshti University of Medical Sciences, Tehran, Iran; 3 Prevention of Metabolic Disorders Research Center, Research Institute for Endocrine Sciences, Shahid Beheshti University of Medical Sciences, Tehran, Iran

**Keywords:** Cyproterone compound, Phlebotomy, Polycystic ovarian syndrome, Randomized control trial (RCT)

## Abstract

**Background:**

Reduction of the body iron stores can improve hyperandrogenemia and insulin resistance. This study aimed to compare clinical and para-clinical responses to the treatment of phlebotomy using oral contraceptive pills (OCs) containing cyproterone acetate in women with PCOS.

**Methods:**

In this randomized clinical trial, 64 patients with PCOS were randomly assigned to the phlebotomy and OCs groups (*n* = 32 in each group). The intervention group, using a single treatment procedure, underwent venesection of 450 mL of whole blood at the early follicular phase of the spontaneous or progesterone-induced menstrual cycle. The control group received OCs pills for 3 months from the 1th day of spontaneous or progesterone-induced menstrual cycle onwards for 3 weeks, followed by a pill-free interval of 7 days. The women were evaluated after the 3-month intervention. The primary outcome measure was a change in the HOMA-IR and free androgen index (FAI). Secondary outcomes were changes in the Ferriman-Gallwey (FG) score and other clinical, biochemical and hormonal changes from the baseline (pre-treatment) to week 12.

**Results:**

In the phlebotomy group, 27 (84.3%) and in the OCs group 30 (93.7%) of the women completed the 3-month follow-up. The median HOMA-IR significantly decreased from 3.5 to 2.7 in the phlebotomy, and from 3.1 to 2.8 in the OCs group, and the changes were comparable between the groups. Median changes in the FAI significantly decreased in both groups, but the differences were not statistically significant between the groups (*P* = 0.061). With regard to secondary outcomes, mean FG scores in both groups significantly decreased [from 16.8 (6) to 13.3 (7.4), *P* < 0.028] in the phlebotomy group and [from 14.3 (7) to 9.8 (7.6) in the OCs group, *P* = 0.001] after 3 months of treatment, but such changes had no statistically significant differences between the groups. During treatment, menstrual cycles became regular in all women in the OCs group and in 12.27 (44.4%) of the women in the phlebotomy group, and the difference was statistically significant (*P* = 0.001). Despite no statistically significant differences in lipid profiles between the groups at the baseline, triglycerides were significantly higher in the OCs group compared to the phlebotomy at end of follow up (*p* = 0.019).

**Conclusion:**

Both treatment modalities had similar beneficial effects on insulin resistance and on androgenic profiles. However, OCs was reported more effective in treating menstrual irregularities and phlebotomy had less adverse effects on triglyceride concentrations.

**Trial registration:**

Code: IRCT2013080514277N1.

## Background

Polycystic ovarian syndrome (PCOS) is the most common ovarian endocrinopathy affecting 6–14% of reproductive aged women [1]. It is characterized by ovulatory dysfunction, clinical or para-clinical hyperandrogenism, and polycystic ovary morphology [2]. Insulin resistance (IR) and obesity are closely associated with PCOS and further reinforce the severity of clinical manifestations [3–5]. Treatment of PCOS is symptom-based and aims to reduce the metabolic risk associated with HA, obesity, and insulin resistance [6–9].

Combined oral contraceptive pills (OCs) have been the key component of relieving PCOS symptoms specifically menstrual irregularity, hirsutism, and acne through improving androgen excess and regulating menstruation [10, 11]. However, there are doubts concerning their impacts on the cardio-metabolism outcomes, which may be aggravated or even triggered by the use of OCs [12, 13]. Nevertheless, the benefits of OCs outweigh related risks in women with PCOS. Since patients with PCOS mostly use these drugs for several years [11], safer therapeutic options are considered suitable for the treatment of PCOS symptoms.

PCOS is associated with iron overload and, which can be associated with IR and further glucose intolerance [14–17]. Factors contributing to potential iron overload in women with PCOS are (i) the iron saved secondary to chronic infrequent menstrual bleeding and (ii) compensatory hyperinsulinemia following insulin resistance. It is believed that insulin favors the intestinal and tissue deposition of iron and can decrease hepcidin, that consequently leads to elevated iron absorption [14, 17, 18]. In addition, reduction of body iron stores can improve hyperandrogenemia and adverse cardio-metabolic effects [19–21]. In earlier studies, iron-chelating agents and blood donation can prevent the development of diabetes due to iron overload [22, 23]. Other studies also have shown that depleting iron stores in diabetes through phlebotomy and blood donation can decrease insulin resistance [24, 25].

The effects of iron reductive therapy in the treatment of PCOS symptoms have not been previously addressed. Therefore, this randomized controlled trial was conducted to investigate the effect of phlebotomy on clinical and biochemical parameters in women with PCOS compared to oral contraceptives containing cyproterone acetate.

## Materials and methods

This parallel non-inferiority randomized controlled trial was conducted on women with PCOS to compare the usual PCOS therapy using OCs containing cyproterone acetate and phlebotomy as the assumed potential modality.

This clinical trial was approved by the ethics committee of the Research Institute of Endocrine Sciences, Shahid Beheshti University of Medical Sciences (decree code: 17ECRIES91/12/08). The research protocol was registered on the Iranian Registry of Clinical Trials at www.irct.ir (decree code: IRCT2013080514277N1). The written informed consent form was signed by the women prior to conducting the study.

### Participants

They were known cases of PCOS based on the Rotterdam criteria [26] and aged 18–45 years, who referred by the gynecologist (FRT) and had at least two of the following criteria: i: oligo/anovulation (defined as either regular or irregular menstruation ≥34 days or history of eight or fewer menstrual cycles in a year); ii: clinical symptoms of hyperandrogenism (including hirsutism diagnosed based on a standardized scoring system of modified Ferriman-Gallwey ≥8, acne and androgenic alopecia) or biochemical hyperandrogenism (defined as the increase of one or more serum level of androgens including testosterone or androstenedione above the 95th percentile, presented in the selected healthy non-hirsute eumenorrheic women of the study population) [27]; iii: polycystic ovaries (having polycystic ovaries with 12 or more follicles in each ovary, 2–9 mm in diameter and/or increased ovarian volume (10 cm^3^)) and exclusion of other etiologies. The women with PCOS with the following criteria were excluded: i) clinically significant organic diseases including malignancy; ii) history of chronic disorders such as diabetes, cardiovascular diseases, hypertension, thyroid disorders, hepatic dysfunction or renal disorder; iii) history of hemochromatosis or presence of the Cys282Tyr mutation; iv) history of smoking and drug or alcohol misuse; and v) history of disturbances in iron balance e.g. iron overload or deficiency; vi) presence of anemia (hemoglobin < 12 g/dL) or serum ferritin < 30 ng/mL.

At baseline hemoglobin, ferritin, 75 g oral glucose tolerance test (OGTT), and the lipid panel were evaluated.

### Randomization

**Fig. 1:**
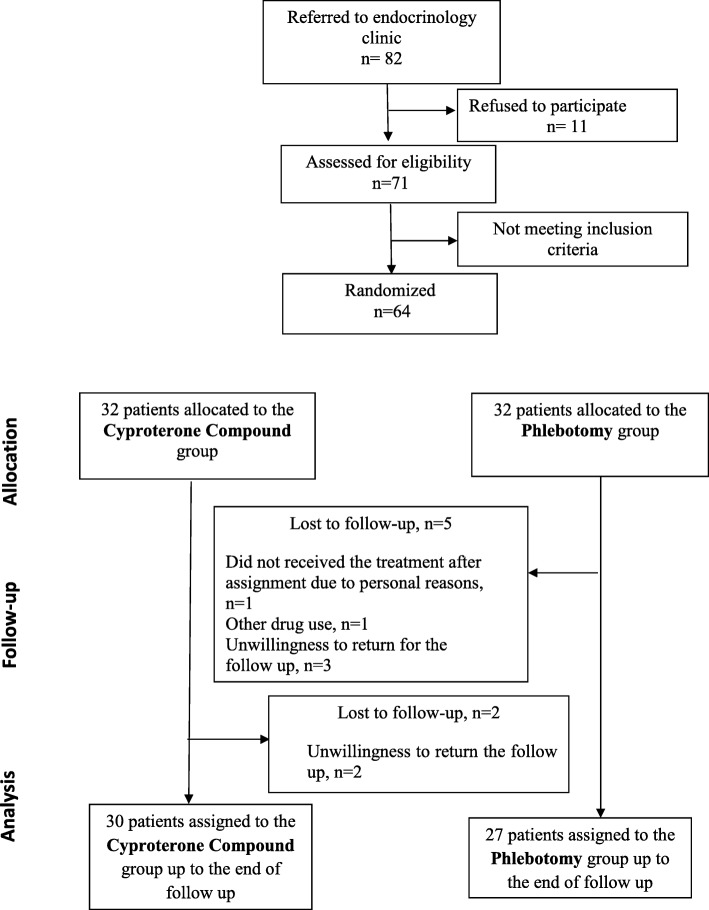
The Consort flow diagram

The process of study has been shown in Fig. 1. Initially, of 82 women with PCOS that were invited to participate in the study, 11 women did not accept it. Of 71 participants, 7 women did not meet the inclusion criteria. Finally 64 women were randomly allocated in a 1:1 ratio to either the iron-reduction phlebotomy group and the OCs containing cyproterone acetate group (*n* = 32 in each group) using a computer-generated randomization table after signing the written informed consent form. Allocation of treatment was not blinded. All participants were asked not to use any medication for PCOS for 6 weeks, but they were advised to maintain their usual diet and physical activity, and also abstain from any other new treatments for PCOS. They referred for phlebotomy to the “Blood Transfusion Unit of Tajrish Hospital”, which was performed by a trained technician under the supervision of a general practitioner.

The study group, in a single treatment procedure, underwent venesection of 450 mL of whole blood (equivalent to 200–220 mL of red blood cells), using a collection bag (Fenwal Europe®, SPRL, Belgium) during the early follicular phase of spontaneous or progesterone-induced menstrual cycle. To prevent volume imbalances, the bloodletting was conducted slowly over 10 min and physiological saline was administered in compensation. The administered volume was equivalent to that of the blood removed. The control group received OCs (35 μg ethinyl estradiol and 2 mg cyproterone acetate; Aburaihan pharmaceutical company®) for 3 months from day the 1th day of a spontaneous or progesterone-induced menstrual cycle onwards for 3 weeks followed by a pill-free interval of 7 days.

It should be noted that the adverse effects of the treatments were monitored via telephone calls in a weekly manner and at the final stage through interviewing.

### Outcome of interest

The primary outcome measure was a change in the HOMA-IR and free androgen index (FAI). Secondary outcomes were changes in the freeman-Galway score and other clinical, and biochemical and hormonal changes from the baseline (pre-treatment) to week 12.

### Clinical and biochemical measurements

Clinical and laboratory assessment was performed before and after the treatment by a trained single staff. In the standing position and with participants wearing minimal clothes, weight and height were measured to the nearest 100 g, following standardized procedures and using calibrated equipment. The waist circumference (WC) was measured midway between the lower rib margin and the iliac-crest at the level of umbilicus and at the end of a gentle expiration. The hip circumference (HC) was measured using an unstretched measuring tape to the nearest 0.1 cm, following which the waist to hip ratio (WHR) was calculated. The body mass index (BMI) was calculated through dividing weight (kg) by the square of height (m).

Systolic and diastolic blood pressure were assessed in the sitting position using a standardized mercury sphygmomanometer which was calibrated by the Iranian Institute of Standards and Industrial Researches.

Fasting blood samples were collected after 10–12 h of fasting. Insulin was measured by Electrochemiluminescence (ECLIA) (Roche Diagnostics GmbH, Mannheim®, Germany). HOMA-IR was calculated by the following formula: [glucose (nmol/L) × insulin (μU/mL)/22.5]. Total cholesterol (TC), high-density lipoprotein cholesterol (HDL-C), and triglycerides were assayed using the enzymatic colorimetric method. Analyses were performed using related kits (Pars Azmon® Inc., Tehran, Iran) and a Selecta 2 autoanalyzer (Vital Scientific®, Dieren, Netherlands). To calculate LDL-C, a modified Friedewald equation was used [28]. The intra- and inter assay coefficients of variation (CV) were: 2.2 and 2.9 for FBS, 0.7 and 2.8 for TG, 2.1 and 2.2 for TC, 2.1 and 2.8 for HDL-C, 1.7 and 1.7 for LDL-C, respectively. The anti-mullerian hormone (AMH) by the two-site enzyme immunoassay method using Gen II kit (Beckman Coulter®, Inc., Fullerton, California) and the Sunrise ELISA reader (Tecan Co®, Salzburg, Austria). Total prostate specific antigen (PSA) levels were assessed by the Electrochemiluminescence (ECLIA) (Roche Diagnostics GmbH®, Mannheim, Germany). Total testosterone were measured by the Electrochemiluminescence (ECLIA) (Roche Diagnostics GmbH®, Mannheim, Germany). Androstenedione and SHBG were measured using the enzyme-linked immunosorbent assay (ELISA) (Pars Azmun Co®. Tehran, Iran). The intra and inter assay coefficients of variation (CV) were: 4 and 4.1 for tT, 3.4 and 3.6 for androstenedione, 3.4 and 5 for SHBG, respectively. The FAI was calculated by the following formula: TT/SHBG × 100 [29].

### Sample size estimation and data analysis

No data from controlled trials were available for estimating of the effect of phlebotomy in women with PCOS; however in a previous trial conducted on the effect of iron reduction on insulin sensitivity, as measured by an intravenous insulin tolerance test in patients with type 2 diabetes, a standardized effect of d = 0.78 was verified [30]. Since the intravenous insulin tolerance test is more sensitive than the HOMA-IR, a hypothetical estimated effect size (mean differences) of 0.2, standard deviation of 0.47 and non-inferiority margin of 0.5 for HOMA-IR as the primary outcome was considered in this study. Under the non-inferiority assumption for this trial, 64 patients (*n* = 32 per group) could provide at least 80% power at a one-sided significance level of 0.05.

An intention to treat analysis was performed and baseline data of the women who had not completed the study process were used as final data.^20^ Related statistics were based on the total number of recruited patients.

Descriptive values were presented as mean (SD) or number (percentage). Distribution of data was assessed using the Kolmogorov–Smirnov test for normality. The t-test and nonparametric were used based on the normal distribution of data. Demographic data and baseline characteristics between the groups were compared using the χ 2 test, t-test, or Mann-Whitney U test. The paired t-test or the Wilcoxon Signed Ranks test were utilized to compare measurements between the baseline and follow-up examinations. Differences between the intervention and control groups in the changes from the baseline to the follow-up examinations were assessed using the t-test or Mann-Whitney U test. All statistical analyses were performed using the SPSS version 11.5 software for Windows (SPSS Inc., Chicago, IL, USA). A significance level of < 0.05 was used during the data analysis.

## Results

In the phlebotomy group, 27 (84.3%) and in the OCs group 30 (93.7%) of the women completed the 3-month follow-up. Five women in the phlebotomy group and 2 women in the OCs group withdrew from the study (Fig. 1). However, the basic characteristics of those women who lost to follow were not significantly different from remaining ones.

The demographic characteristics of the women were shown in Table 1. The mean age and BMI in the OCs vs. the phlebotomy group were 24 (4.06) vs. 24.2 (5.2), years and 27.1 (5.04) vs. 25.4 (5.3), kg/m^2^, respectively at the initiation of the study.

**Table 1:** The baseline characteristics of the samples

	Phlebotomy *N* = 27	OCs *N* = 30	*P*-value*
Age, (y)	24 (4.06)	24.2 (5.2)	0.878
BMI, (kg/m^2^)	27.1 (5.04)	25.4 (5.3)	0.323
WHR,	0.8 (0.06)	0.7 (0.08)	0.223
HB, (g/dl)	14.1 (1.1)	14.3 (1.3)	0.166
HCT	40.4 (2.1)	39.6 (2.3)	0.330
Ferritin, (ng/mL)	74.2 (54.06)	48.5 (30.9)	0.070
SBP, (mm Hg)	102.3 (9.0)	100.2 (18.9)	0.526
DBP, (mm Hg)	67.6 (6.4)	66.2 (7.8)	0.503
Hirsutism score,	16.8 (6)	14.3 (7)	0.063
Acne, yes (%)	13 (74)	17 (56.6)	0.189
FAI, median	5.2 (1.6–12.8)	4.4 (1.4–4.7)	0.051
Total Testosterone, (ng/mL)	0.52 (0.3)	0.40 (0.1)	0.095
Androstenedione, (ng/mL)	4.7 (1.9)	4.1 (1.8)	0.350
SHBG, (nmol/L)	35.5 (20.9)	46.6 (46.8)	0.059
HOMA-IR, median	3.5 (1.3–4.5)	3.1 (1.4–3.5)	0.595
Insulin, (μU/mL)	9.5 (6.8–20)	10.8 (7.6–14.9)	0.626
GTT, fasting, (mg/dL)	84.3 (6.6)	83.8 (6.7)	0.813
GTT, 2 h, (mg/dL)	101.9 (12.7)	90.2 (23.9)	0.940
Cholesterol, (mg/dL)	178.5 (37.9)	178.7 (33.3)	0.985
Triglycerides, (mg/dL)	115.5 (107.9)	114.2 (48)	0.957
HDL-C, (mg/dL)	45.5 (15.1)	47.5 (10.3)	0.611
LDL-C, (mg/dL)	95.5 (26)	97.1 (22.7)	0.831
AMH, (ng/mL)	8.1 (6)	8.7 (6)	0.471
PSA, (ng/mL)	0.005 (0.002–0.009)	0.004 (0.002–0.020)	0.688

*The statistical significance level was set at 0.05; Values are given as mean (standard deviation) or median (25–75%), as appropriate.

*BMI* Body Mass Index; *WHR* Waist to Hip Ratio; *HB* Hemoglobin; *HCT* Hematocrit; *SBP* Systolic blood pressure; *DBP* Diastolic blood pressure; *FAI* Free Androgen Index; *SHBG* Sex Hormone Androgen Index; *HOMA-IR* Homeostatic Model Assessment for Insulin Resistance; *GTT* Glucose tolerance test; *HDL* High Density Lipoprotein; *LDL* Low Density Lipoprotein; *AMH* Anti Mullerian Hormone; *PSA* Prostate Specific Antigen.; *SHBG* Sex hormone-binding globulin; *OCs* oral contraceptives containing cyproterone acetate

At baseline, all parameters were comparable between the groups and no statistically significant differences between the groups with regard to age, BMI, androgenic profile, and other carbohydrate or metabolic profile were reported.

Prior to the treatment, 77.7% (21 of 27) of the phlebotomy group and 80% (24 of 30) of the OCs group had oligo-menorrhea or amenorrhea. While all women in the OCs group had a normal menstruation after treatment, but 12/27 (44.4%) in the phlebotomy group still suffered from oligo-menorrhea or amenorrhea, which was statistically significant (*P* = 0.002).

Effects of both treatments on HOMA-IR were presented in Table 2. A statistically significant decline of HOMA-IR from the baseline compared to the end of the 3th month of the treatment in both groups [3.5 (1.3–4.5) vs. 2.7 (1.2–4.3), *P* = 0.047 and 3.1 (1.4–3.5) vs. 2.8 (1.8–3.9), *P* = 0.032, in the phlebotomy and the OCs groups, respectively] was reported, but it was not statistically significant (*P* = 0.516).

**Table 2:** Effects of the treatment in the groups

	Phlebotomy *N* = 27		*P*-value*	OCs *N* = 30		*P*-value*	*P*-value**
	3 months post-treatment	Mean difference		3 months post-treatment	Mean difference		
BMI	27 (4.7)	−0.01	0.229	25.1 (5.3)	−0.03	0.784	0.355
WHR	0.8 (0.04)	−0.01	0.346	0.7 (0.05)	−0.01	0.621	0.412
Hirsutism score	13.3 (7.4)	−3.7 (6.1)	0.028	9.8 (7.6)	−2.1 (2.8)	0.001	0.235
SBP, (mm Hg)	101.1 (6.5)	−1.1 (10.3)	0.647	102 (8.4)	1.5 (20.1)	0.690	0.610
DBP, (mm Hg)	65.8 (7.7)	−1.7 (10.8)	0.514	65.8 (5.3)	−0.5 (10)	0.780	0.701
HOMA_IR	2.7 (1.2–4.3)	−0.73 (2)	0.047	2.8 (1.8–3.9)	−0.45 (5.6)	0.032	0.516
Insulin, (μU/mL)	8.1 (6.5–15.7)	−0.5 (10.1)	0.025	9.8 (7.8–16.1)	−4.6 (23.8)	0.015	0.512
FAI	4.1 (1.8–8)	−1.1 (3.4)	0.042	3.1 (0.3–6.8)	−1.2 (2.9)	0.001	0.061
Total Testosterone, (ng/mL)	0.42 (0.34)	−0.07 (0.1)	0.047	0.33 (0.1)	−0.06 (0.1)	0.045	0.378
Androstenedione, (ng/mL)	3.3 (1.7)	−1.3 (1.3)	0.026	2.9 (1.6)	−1.1 (1.8)	0.001	0.070
SHBG, (nmol/L)	91.1 (72.4)	55.6 (63)	0.013	163.7 (101.5)	107.06 (115.4)	0.001	0.009
Cholesterol, (mg/dL)	179.8 (67.1)	1.2 (69.4)	0.940	183.2 (41)	3.4 (32.5)	0.076	0.102
Triglycerides, (mg/dL)	123.8 (93.4)	8.3 (83.9)	0.687	152 (89.9)	37.7 (65.1)	0.004	0.019
HDL, (mg/dL)	43 (15.6)	−2.5 (13.9)	0.467	51.9 (15)	4.4 (11.2)	0.053	0.072
LDL, (mg/dL)	93.4 (27)	−2.1 (22.9)	0.709	105.9 (33.4)	8.8 (32.2)	0.150	0.187
AMH, (ng/mL)	8 (5.4)	−0.1 (3.6)	0.909	6.7 (3.8)	−1.9 (5)	0.043	0.191
PSA, (ng/mL)	0.002 (0.002–0.008)	0.003 (0.01)	0.427	0.002 (0.002–0.01)	−0.005 (0.01)	0.204	0.058

Comparison of the parameters with baseline **Comparison of mean differences

Values are given as mean (standard deviation) or median (25%-75%), as appropriate

*SBP* Systolic blood pressure; *DBP* Diastolic blood pressure; *FAI* Free Androgen Index; *SHBG* Sex Hormone Androgen Index; *HOMA-IR* Homeostatic Model Assessment for Insulin Resistance; *HDL* High Density Lipoprotein; *LDL* Low Density Lipoprotein; *AMH* Anti Mullerian Hormone; *PSA* Prostate Specific Antigen; *OCs* oral contraceptives containing cyproterone acetate

Both treatments significantly decreased insulin levels throughout the study (9.5 (6.8–20) vs. 8.1 (6.5–15.7) *P* = 0.025 and 10.8 (7.6–14.9) vs. 9.8 (7.8–16.1), *P* = 0.015 in the phlebotomy and the OCs group, respectively). Moreover, mean differences were not statistically significant between the groups.

All androgenic parameters including testosterone, androstenedione and FAI decreased in both groups. However, no statistically significant differences between the groups were reported (Table 2). Compared to the baseline, SHBG showed a rising trend during the treatment in both groups, but the mean score of changes in the OCs was significantly higher than of the phlebotomy groups (*P* = 0.009).

Despite no statistically significant difference in lipid profiles of both groups at the initiation of study (Table 1), there was a statistically significant rise in triglycerides from the baseline in the OCs (114.2 (48) vs. 152 (89.9), *P* = 0.004) at the end of the 3th month of the treatment. Accordingly, the mean score changes of TG between the groups was statistically significant (8.3 (83.9) vs. 37.7 (65.1), *P* = 0.019). Changes in LDL-C, HDL-C and TC had no statistically significant differences within and between the groups.

At the baseline, the mean FG scores in the phlebotomy and the OCs groups were 16.8 (6) and 14.3 (7), respectively as decreased to 13.3 (7.4), *P* = 0.001 and 9.8 (7.6), *P* = 0.028, respectively, However, the changes were not statistically significant between the groups (Table 2). AMH and PSA remained unchanged in both groups throughout the study.

### Safety

Phlebotomy were well-tolerated by all women and no serious side effects were reported. Dizziness and headaches for a few hours occurred in five women after phlebotomy, but they consented to undergo repeated phlebotomies, if needed.

## Discussion

This is the first study on the endocrine, clinical and metabolic effects of treatment using phlebotomy in women with PCOS and comparison of the beneficial effects of two different treatments using the OCs containing cyproterone acetate and phlebotomy. As a result, both treatments had similar beneficial effects on insulin resistance as well as on androgenic profiles. However, OCs containing cyproterone acetate was found to be more effective in the treatment of menstrual irregularity and phlebotomy and had less adverse effects.

Ethinyl estradiol and cyproterone acetate in combination (cyproterone compound) have been widely used as reliable contraceptive pills in women with PCOS suffering from hyperanderogenism [31]. Such a combination can improve various PCOS manifestations through several potential mechanisms including the inhibition of LH secretion from the pituitary, reduction of androgen production from ovaries, reduction of free androgens, increase of circulating SHBG levels and thereby limiting peripheral androgen exposure. Cyproterone acetate can block peripheral androgen receptors at target organs and can reduce the ovarian androgen production and decrease the plasma levels of free testosterone [32].

However, OCs are known to have adverse effects on lipid metabolism and carbohydrate intolerance. Estrogen has pro-thrombotic effects and increases the cardiovascular venous thromboembolism [33].

The increased potential adverse cardio-metabolic effects of these drugs encourage the use of non-pharmacological treatments for relieving PCOS symptoms. Surprisingly, a lower prevalence of diabetes has been observed among blood donors [34]. Recently, it has been shown that hyperandrogenemia and insulin resistance are associated with ferritin [35, 36].

Emerging evidence hence suggests that in the general population, body iron stores and iron intake are positively associated with glucose intolerance and diabetes [21, 37, 38]. Although, potential underlying mechanisms are not fully understood, iron influences insulin production and sensitivity, and together they influence on iron metabolism. Therefore, iron interferes with the insulin prohibition of hepatic glucose secretion, and hepatic iron stores reduce the production of insulin leading to systemic hyperinsulinemia [14]. In this respect, previous studies found the beneficial effects of phlebotomy and blood donation in patients with type 2 diabetes [24, 25, 39], demonstrating that iron reduction by phlebotomy improved insulin sensitivity.

Women with PCOS may suffer from iron overload due to chronic infrequent menstrual bleeding, that in turn reduces menstrual blood loss along with reduced serum hepcidin levels as an iron-regulatory hormone [40]. It is also affected by insulin resistance and hyperandrogenism that may improve iron absorption and reduce iron secretion from macrophages, and possibly leads to mild iron overload in some patients with PCOS [17, 35, 41]. Such an overload can contribute to glucose intolerance in women with PCOS [35] independent of chronic inflammation [42].

Consistent with this hypothesis, a few studies are available on the effects of therapeutic interventions on iron exess in women with PCOS. In this respect, Luque-Ramírez et al. (2011) in a randomized clinical trial compared the effect of metformin as an insulin sensitizer with an antiandrogenic contraceptive pill for 6 months and found that patients with PCOS decreased hepcidin levels that might contribute to iron overload through facilating the intestinal absorption of iron [40].

However, our study as a pioneer investigation confirmed that phlebotomy enhanced insulin sensitivity and improved the state of hyperanderogenism in women with PCOS. However, more studies with longer follow-ups are still needed to confirm these thraputic effects.

In the present study, the side effects of phlebotomy were rare. The appropriate selection of vein and careful procedure of venipuncture helped us to minimize probable vasovagal reactions.

As the limitation of this study the short study duration might not have shown the long terms effect of this treatment, because the improvement of some PCOS manifestations might need long term follow-ups. In addition, some PCOS confounders including the women lifestyle were not assessed. No enogh power was present to performe the subgroup analysis based on various PCOS phenotypes or obesity status. Also, the HOMA-IR has been used as a surrogate marker for the assessment of IR, despite the good correlation between HOMA-IR and gold standard clamp methods [43], which might be inaccurate in women with PCOS [44]. Also, this study did not measure free testosterone due to a lack of access to the proper method. It has been shown that FAI has a good correlation with free testosterone [45]. Since ferritin was not measured at the end of the study follow-up, assessment of changes in iron overload was impossible. Moreover, the number of lost to follow up subjects in the phlebotomy group was higher than those in the OCs group, but our study results may not be affected as there would be no statistically significant differences in basic characteristics between those who lost to follow up and the remained participants in each group. In addition, we cross validated our data and found that the internal validity of the results was not affected by lost to follow up. Finally, the sample size was calculated based on the HOMA-IR, and there was not adequate number of subjects for the precise comparison of other clinical or para-clinical manifestations of PCOS. Therefore, observiation of no significant differences on these items could be due to the small sample size, which should be interpreted with cautious.

## Conclusion

This study suggest that the phlebotomy therapy is associated with a tendency to decrease insulin resistance and hyperanderogenism, that merits further investigations. In addition, large prospective, randomized controlled trials with longer treatment periods are recommended to confirm the study findings.

## Data Availability

The datasets used and analyzed during the current study are available via sending email to the corresponding author in case of request for future research.

## Abbreviations

AMH: Anti-mullerian hormone
BMI: body mass index
DHEAS: Dehydroepiandrosterone sulfate
FAI: Free androgen index
FG: Ferriman-Gallwey
HC: Hip circumference
HDL-C: high density lipoprotein-cholesterol
HOMA-IR: Homeostatic Model Assessment for Insulin Resistance
LDL-C: low density lipoprotein-cholesterol
OCPs: Combined oral contraceptive pills
OCs: oral contraceptive pills
PCOS: Polycystic ovarian syndrome
PSA: prostate specific antigen
SD: standard deviation
SHBG: Sex hormone-binding globulin
TC: Total cholesterol
TT: total testosterone
WC: waist circumference
WHR: waist to hip ratio

## References

[1] Tehrani FR, Simbar M, Tohidi M, Hosseinpanah F, Azizi F (2011). The prevalence of polycystic ovary syndrome in a community sample of Iranian population: Iranian PCOS prevalence study. Reprod Biol Endocrinol.

[2] Sirmans SM, Pate KA (2014). Epidemiology, diagnosis, and management of polycystic ovary syndrome. Clin Epidemiol.

[3] Behboudi-Gandevani S, Ramezani Tehrani F, Rostami Dovom M, Farahmand M, Bahri Khomami M, Noroozzadeh M (2016). Insulin resistance in obesity and polycystic ovary syndrome: systematic review and meta-analysis of observational studies. Gynecol Endocrinol.

[4] Behboudi-Gandevani S, Amiri M, Bidhendi Yarandi R, Noroozzadeh M, Farahmand M, Rostami Dovom M (2018). The risk of metabolic syndrome in polycystic ovary syndrome: a systematic review and meta-analysis. Clin Endocrinol.

[5] Rostami Dovom M, Ramezani Tehrani F, Djalalinia S, Cheraghi L, Behboudi Gandavani S, Azizi F (2016). Menstrual cycle irregularity and metabolic disorders: a population-based prospective study. PLoS One.

[6] Mirza SS, Shafique K, Shaikh AR, Khan NA, Anwar QM (2014). Association between circulating adiponectin levels and polycystic ovarian syndrome. J Ovarian Res.

[7] Kazemi Jaliseh H, Ramezani Tehrani F, Behboudi-Gandevani S, Hosseinpanah F, Khalili D, Cheraghi L (2017). Polycystic ovary syndrome is a risk factor for diabetes and prediabetes in middle-aged but not elderly women: a long-term population-based follow-up study. Fertil Steril.

[8] Amiri M, Ramezani Tehrani F, Nahidi F, Bidhendi Yarandi R, Behboudi-Gandevani S, Azizi F (2017). Association between biochemical hyperandrogenism parameters and Ferriman-Gallwey score in patients with polycystic ovary syndrome: a systematic review and meta-regression analysis. Clin Endocrinol.

[9] Behboudi-Gandevani S, Ramezani Tehrani F, Bidhendi Yarandi R, Noroozzadeh M, Hedayati M, Azizi F (2017). The association between polycystic ovary syndrome, obesity, and the serum concentration of adipokines. J Endocrinol Investig.

[10] Mendoza N, Simoncini T, Genazzani AD (2014). Hormonal contraceptive choice for women with PCOS: a systematic review of randomized trials and observational studies. Gynecol Endocrinol.

[11] Yildiz BO (2015). Approach to the patient: contraception in women with polycystic ovary syndrome. J Clin Endocrinol Metab.

[12] Harmanci A, Cinar N, Bayraktar M, Yildiz BO (2013). Oral contraceptive plus antiandrogen therapy and cardiometabolic risk in polycystic ovary syndrome. Clin Endocrinol.

[13] de Melo AS, Dos Reis RM, Ferriani RA, Vieira CS (2017). Hormonal contraception in women with polycystic ovary syndrome: choices, challenges, and noncontraceptive benefits. Open Access J Contracept.

[14] Tiongco RE, Rivera N, Clemente B, Dizon D, Salita C, Pineda-Cortel MR (2019). Serum ferritin as a candidate diagnostic biomarker of polycystic ovarian syndrome: a meta-analysis. Biomarkers.

[15] Escobar-Morreale HF (2012). Iron metabolism and the polycystic ovary syndrome. Trends Endocrinol Metab.

[16] Escobar-Morreale HF1, Luque-Ramírez M, Alvarez-Blasco F, Botella-Carretero JI, Sancho J, San Millán JL (2005). Body iron stores are increased in overweight and obese women with polycystic ovary syndrome. Diabetes Care.

[17] Luque-Ramírez M, Alvarez-Blasco F, Botella-Carretero JI, Sanchón R, San Millán JL, Escobar-Morreale HF (2007). Increased body iron stores of obese women with polycystic ovary syndrome are a consequence of insulin resistance and hyperinsulinism and are not a result of reduced menstrual losses. Diabetes Care.

[18] Stechemesser L, Eder SK, Wagner A, Patsch W, Feldman A, Strasser M (2016). Metabolomic profiling identifies potential pathways involved in the interaction of iron homeostasis with glucose metabolism. Mol Metab.

[19] Houschyar KS, Lüdtke R, Dobos GJ, Kalus U, Broecker-Preuss M, Rampp T (2012). Effects of phlebotomy-induced reduction of body iron stores on metabolic syndrome: results from a randomized clinical trial. BMC Med.

[20] Valenti L, Fracanzani AL, Dongiovanni P, Bugianesi E, Marchesini G, Manzini P (2007). Iron depletion by phlebotomy improves insulin resistance in patients with nonalcoholic fatty liver disease and hyperferritinemia: evidence from a case-control study. Am J Gastroenterol.

[21] Bao W, Rong Y, Rong S, Liu L (2012). Dietary iron intake, body iron stores, and the risk of type 2 diabetes: a systematic review and meta-analysis. BMC Med.

[22] Olivieri NF, Brittenham GM (1997). Iron-chelating therapy and the treatment of thalassemia. Blood.

[23] Dmochowski K, Finegood DT, Francombe W, Tyler B, Zinman B (1993). Factors determining glucose tolerance in patients with thalassemia major. J Clin Endocrinol Metab.

[24] Equitani F, Fernandez-Real JM, Menichella G, Koch M, Calvani M, Nobili V (2008). Bloodletting ameliorates insulin sensitivity and secretion in parallel to reducing liver iron in carriers of HFE gene mutations. Diabetes Care.

[25] Fernández-Real JM, Peñarroja G, Castro A, García-Bragado F, López-Bermejo A, Ricart W (2002). Blood letting in high-ferritin type 2 diabetes: effects on vascular reactivity. Diabetes Care.

[26] Rotterdam ESHRE/ASRM-Sponsored PCOS Consensus Workshop Group (2004). Revised 2003 consensus on diagnostic criteria and long-term health risks related to polycystic ovary syndrome. Fertil Steril.

[27] Hashemi S, Ramezani Tehrani F, Noroozzadeh M, Azizi F (2014). Normal cut-off values for hyperandrogenaemia in Iranian women of reproductive age. Eur J Obstet Gynecol Reprod Biol.

[28] Chen Y, Zhang X, Pan B, Jin X, Yao H, Chen B (2010). A modified formula for calculating low-density lipoprotein cholesterol values. Lipids Health Dis.

[29] Wilke TJ, Utley DJ (1987). Total testosterone, free-androgen index, calculated free testosterone, and free testosterone by analog RIA compared in hirsute women and in otherwise-normal women with altered binding of sex-hormone-binding globulin. Clin Chem.

[30] Fernández-Real JM, Peñarroja G, Castro A, García-Bragado F, Hernández-Aguado I, Ricart W (2000). Blood letting in high-ferritin type 2 diabetes. Effects on Insulin Sensitivity and β-Cell Function. Diabetes.

[31] Ruan X, Kubba A, Aguilar A, Mueck AO (2017). Use of cyproterone acetate/ethinylestradiol in polycystic ovary syndrome: rationale and practical aspects. Eur J Contracept Reprod Health Care.

[32] Gode F, Karagoz C, Posaci C, Saatli B, Uysal D, Secil M (2011). Alteration of cardiovascular risk parameters in women with polycystic ovary syndrome who were prescribed to ethinyl estradiol–cyproterone acetate. Arch Gynecol Obstet.

[33] Shufelt CL1, Bairey Merz CN (2009). Contraceptive hormone use and cardiovascular disease. J Am Coll Cardiol.

[34] Ascherio A, Rimm EB, Giovannucci E, Willett WC, Stampfer MJ (2001). Blood donations and risk of coronary heart disease in men. Circulation.

[35] Martínez-García MA, Luque-Ramírez M, San-Millán JL, Escobar-Morreale HF (2009). Body iron stores and glucose intolerance in premenopausal women: role of hyperandrogenism, insulin resistance and genomic variants related to inflammation, oxidative stress and iron metabolism. Diabetes Care.

[36] Brudevold R, Hole T, Hammerstrøm J (2008). Hyperferritinemia is associated with insulin resistance and fatty liver in patients without iron overload. PLoS One.

[37] Swaminathan S, Fonseca VA, Alam MG, Shah SV (2007). The role of iron in diabetes and its complications. Diabetes Care.

[38] Montonen J, Boeing H, Steffen A, Lehmann R, Fritsche A, Joost H (2012). Body iron stores and risk of type 2 diabetes: results from the European prospective investigation into Cancer and nutrition (EPIC)-Potsdam study. Diabetologia.

[39] Bofill C, Joven J, Bages J, Vilella E, Sans T, Cavallé P (1994). Response to repeated phlebotomies in patients with non-insulin-dependent diabetes mellitus. Metabolism.

[40] Luque-Ramírez M, Álvarez-Blasco F, Alpañés M, Escobar-Morreale HF (2011). Role of decreased circulating hepcidin concentrations in the iron excess of women with the polycystic ovary syndrome. J Clin Endocrinol Metab.

[41] Lecube A, Hernández C, Pelegrí D, Simó R (2008). Factors accounting for high ferritin levels in obesity. Int J Obes.

[42] Escobar-Morreale HF, Luque-Ramírez M, San Millán JL (2005). The molecular-genetic basis of functional hyperandrogenism and the polycystic ovary syndrome. Endocr Rev.

[43] Skrha J, Haas T, Sindelka G, Prázný M, Widimský J, Cibula D (2004). Comparison of the insulin action parameters from hyperinsulinemic clamps with homeostasis model assessment and QUICKI indexes in subjects with different endocrine disorders. J Clin Endocrinol Metab.

[44] Lawson MA, Jain S, Sun S, Patel K, Malcolm PJ, Chang RJ (2008). Evidence for insulin suppression of baseline luteinizing hormone in women with polycystic ovarian syndrome and normal women. J Clin Endocrinol Metab.

[45] Rosner W, Auchus RJ, Azziz R, Sluss PM, Raff H (2007). Position statement: utility, limitations, and pitfalls in measuring testosterone: an Endocrine Society position statement. J Clin Endocrinol Metab.

